# Evidence in Obese Children: Contribution of Hyperlipidemia, Obesity-Inflammation, and Insulin Sensitivity

**DOI:** 10.1371/journal.pone.0125935

**Published:** 2015-05-26

**Authors:** Chi-Jen Chang, Deng-Yuan Jian, Ming-Wei Lin, Jun-Zhi Zhao, Low-Tone Ho, Chi-Chang Juan

**Affiliations:** 1 Division of Pediatric Surgery, Department of Surgery, Shin-Kong Wu Ho-Su Hospital, Taipei, Taiwan; 2 School of Medicine, Fu Jen Catholic University, New Taipei City, Taiwan; 3 Institutes of Physiology, School of Medicine, National Yang-Ming University, Taipei, Taiwan; 4 Division of Nephrology, Wen-Lin Hemodialysis Unit, Taipei, Taiwan; 5 Institute of Public Health, School of Medicine, National Yang-Ming University, Taipei, Taiwan; 6 Department of Medical Research and Education, Taipei Veterans General Hospital, Taipei, Taiwan; 7 Department of Education and Research, Taipei City Hospital, Taipei, Taiwan; East Tennessee State University, UNITED STATES

## Abstract

**Background:**

Evidence shows a high incidence of insulin resistance, inflammation and dyslipidemia in adult obesity. The aim of this study was to assess the relevance of inflammatory markers, circulating lipids, and insulin sensitivity in overweight/obese children.

**Methods:**

We enrolled 45 male children (aged 6 to 13 years, lean control = 16, obese = 19, overweight = 10) in this study. The plasma total cholesterol, HDL cholesterol, triglyceride, glucose and insulin levels, the circulating levels of inflammatory factors, such as TNF-α, IL-6, and MCP-1, and the high-sensitive CRP level were determined using quantitative colorimetric sandwich ELISA kits.

**Results:**

Compared with the lean control subjects, the obese subjects had obvious insulin resistance, abnormal lipid profiles, and low-grade inflammation. The overweight subjects only exhibited significant insulin resistance and low-grade inflammation. Both TNF-α and leptin levels were higher in the overweight/obese subjects. A concurrent correlation analysis showed that body mass index (BMI) percentile and fasting insulin were positively correlated with insulin resistance, lipid profiles, and inflammatory markers but negatively correlated with adiponectin. A factor analysis identified three domains that explained 74.08% of the total variance among the obese children (factor 1: lipid, 46.05%; factor 2: obesity-inflammation, 15.38%; factor 3: insulin sensitivity domains, 12.65%).

**Conclusions:**

Our findings suggest that lipid, obesity-inflammation, and insulin sensitivity domains predominantly exist among obese children. These factors might be applied to predict the outcomes of cardiovascular diseases in the future.

## Introduction

Obesity or overweight has become a global epidemic and affects both children and adults [1–3]. Obesity is a major risk factor for insulin resistance in children with metabolic syndrome [4]. Accumulating evidence shows that the increase in childhood obesity and the earlier onset of insulin resistance, hypertension, and dyslipidemia facilitate the development of risk factors for cardiovascular disease [4,5]. These metabolic abnormalities in children may indicate that diabetes and cardiovascular disease complications appear earlier than previously thought.

Obese individuals often present with metabolic disorders, such as high blood pressure, elevated fasting glucose levels and lipid abnormalities, which promote vascular injury and endothelial dysfunction [6]. Highlighting the role of inflammation in obesity, adipose tissue from obese individuals is characterized by inflammation and can secrete humoral factors that regulate systemic acute-phase reactants, such as C-reactive protein (CRP) [7,8], as well as inflammatory factors, such as monocyte chemo-attractant protein-1 (MCP-1) [9], tumor necrosis factor-α (TNF-α) [10], and interleukin-6 (IL-6) [11,12]. Moreover, macrophages are involved in obesity-induced insulin resistance and facilitate obesity-induced inflammation [13]. The inflammation process is required for the initiation and development of atherosclerosis [14]. The levels of inflammatory markers, such as high sensitivity CRP (hs-CRP) [4], TNF-α and IL-6, are high during inflammation and are related to the pathogenesis of cardiovascular disease. These markers have also been shown to correlate with the subsequent development of cardiovascular disease in obese individuals [15,16]. Furthermore, inflammatory cytokine levels, including high TNF-α, high leptin levels, and low adiponectin levels, are associated with insulin resistance in obese children [17–20] and also affect physical activity during the growth and maturation process [21]. Additional research has noted that lifestyle changes can reduce obesity and blood inflammatory marker levels in both children and adolescents [22–24]. Some studies have also directly measured insulin levels and used factor analyses to assess the risk of dangerous values of other metabolic and inflammatory variables in patients with non-type 2 diabetes mellitus as well as to explore the correlation between these risk factors and the development of type 2 diabetes mellitus [25,26].

The pathogenesis of obesity-related atherosclerosis, which is marked by hypoadiponectinemia and high serum levels of leptin and TNF-α in overweight and obese individuals, serves an important function in the initiation of inflammation [27–29]. However, the exact connection between these inflammatory markers and the development of childhood obesity remains unclear. This study aims to evaluate the changes in inflammatory markers, circulating lipid profiles, and insulin sensitivity among overweight and obese children. Furthermore, we seek to clarify the relationships among inflammation, lipid profiles, and insulin sensitivity as well as observe their contribution to metabolic risk using a factor analysis.

## Materials and Methods

### Selection of Patients

Forty-five male children aged six to eighteen years were recruited from one outpatient department of the Taipei Medical Center in Taiwan. In our study, only male children were studied because we wanted to avoid the effects that sex hormones have on obesity. Overweight and obese participants were referred to the pediatric clinic of the author by their general practitioner between 2010 and 2011. Patients were classified as obese if their body mass index (BMI) reached or exceeded the 95th percentile of their age and sex cohorts; children were classified as overweight if their BMI was in the 85th to 95th percentile of their same age and sex cohorts [4,30].

### Ethics Statement

The program moderators provided a detailed explanation of the nature and purpose of the study to participants. Before the participants were enrolled in the experiment, the moderators answered their questions in separate interview rooms. The children agreed to participate in the experiment, and either their parents or their legal representatives provided signed informed consent, unless the subject was 18 years old and thus able to decide for themselves. Each participant has the right to have their data removed from the research at any time according to the principles stated by the ethics committees. The Committees on the Use and Care of Animals and the Human Institutional Review Board (IRB; No. 9809–009) of the Shin-Kong Wu Ho-Su Hospital, Taipei approved this study and its procedures.

### Study Procedures

A medical history was taken, and a physical examination was performed at the clinic of the author. Sitting blood pressure was measured three times using an automated sphygmomanometer (with at least 20 min between blood pressure measurements). A digital scale and a Harpenden stadiometer were used to measure body mass and height, respectively, and these values were used to calculate BMI (kilograms per square meter). Overnight-fasting peripheral blood samples (10 mL) were obtained from overweight subjects, obese subjects, and lean controls, and the hormone, cytokine, substrate, and glucose concentrations were measured, along with a lipid panel.

### Biochemical Analysis

Plasma total cholesterol, HDL cholesterol, triglyceride, glucose, and insulin levels were measured using commercial kits. LDL cholesterol was calculated using the Friedewald formula [31]. Circulating levels of inflammatory factors such as TNF-α, IL-6, MCP-1, and hs-CRP were determined using specific quantitative colorimetric sandwich ELISA kits according to the manufacturer’s instructions. The homeostatic model assessment index (HOMA index = fasting glucose [mmol/L] × fasting insulin [μU/mL] / 22.5) was used as a measure of insulin sensitivity.

### Data Analysis

All values were expressed as the mean and the standard error of the mean (SEM). Differences between groups were evaluated using Student’s t test or one-way analysis of variance where appropriate. The correlations between parameters were calculated using Pearson product moment correlation. All statistical analyses were performed using SPSS 20.0 (IBM Corporation, New York, USA). A P-value of less than 0.05 was considered significant.

In addition, we applied a factor analysis using the principal component method with a Varimax rotation to examine whether fasting insulin level was clustered with the anthropometric, metabolic, and inflammatory variables. BMI percentile, fasting glucose, LDL-c/HDL-c ratio, HDL-c/TC ratio, LDL-c/TC ratio, TC/HDL-c ratio, hs-CRP, TNF-α, and PAI-1 were included as variables in the factor analysis. A factor loading with an absolute value of ± 0.5 or greater was used as a cut-off value for data interpretation. The total variance explained by each factor was presented to indicate the individual effect of the factor in the analysis.

## Results

### The Clinical Characteristics of Enrolled Participants

Forty-five male children were enrolled in this study: 10 were overweight, 19 were obese, and 16 were lean. By definition, the obese and overweight children showed significantly greater body weight (overweight and obese vs. lean, 44.40 ± 1.78 / 49.77 ± 2.71 vs. 36.00 ± 2.39 kilograms; *p*<0.05) and BMI (overweight/obese vs. lean, 90.89 ± 0.75 / 98.06 ± 0.22 vs. 59.79 ± 5.31 percentile; *p*<0.05). No significant differences were found between the obese and lean groups with respect to age, height, systolic blood pressure, diastolic pressure, or mean blood pressure. In addition, diastolic blood pressure was higher in the overweight children than the obese children, but this difference was not significant. The blood pressure difference (ΔBP) between systolic blood pressure (SBP) and diastolic blood pressure (DBP) was significantly lower among the overweight children than the obese children (46.25 ± 4.95 vs. 59.29 ± 2.85; *p*<0.05). The clinical characteristics of the participants are summarized in Table 1.

**Table 1:** The clinical characteristics of forty-five male children at one Taiwanese center.

	Lean	Overweight	Obese
n	16	10	19
Age (yr)	10.06 ± 0.60	10.70 ± 0.37	9.32 ± 0.45
Height (cm)	141.44 ± 3.30	144.20 ± 2.21	141.29 ± 2.43
Weight (kg)	36.00 ± 2.39	44.40 ± 1.78*	49.77 ± 2.71*
BMI (kg/m^2^)	17.63 ± 0.47	21.28 ± 0.37*	24.52 ± 0.53* ^#^
BMI (percentile)	59.79 ± 5.31	90.89 ± 0.75*	98.06 ± 0.22* ^#^
Systolic BP (mm Hg)	115.27 ± 3.39	118.88 ± 3.78	122.57 ± 3.74
Diastolic BP (mm Hg)	57.80 ± 3.05	72.63 ± 6.38	63.29 ± 3.08
ΔBP (mm Hg)	57.47 ± 3.22	46.25 ± 4.95	59.29 ± 2.85^#^
Mean BP (mm Hg)	76.96 ± 2.78	88.04 ± 5.14	83.05 ± 3.03

Values are given as the mean ± SEM. Abbreviations: BMI, body mass index; BP, blood pressure; ΔBP, Systolic BP-Diastolic BP.

* P＜0.05, differences vs. lean.

# P＜0.05, differences vs. overweight.

### Measures of Metabolic and Inflammatory Parameters

The metabolic and inflammatory parameters of the lean, overweight, and obese participants are listed in Table 2. Although no significant difference was observed in the plasma glucose value between the obese and lean children, the fasting glucose, insulin, and HOMA levels were significantly higher among the obese subjects than the lean subjects. These data indicate a significant degree of insulin resistance in the obese children compared with that in the lean control. Compared with the lean children, the obese group showed increased plasma LDL-cholesterol and TG levels and a decreased plasma HDL-cholesterol level. However, no increase in the LDL-cholesterol and TG values was observed in the overweight children, despite the significantly high HDL-cholesterol level in the obese children (Table 2). This finding showed that the obese subjects had higher levels of the various components measured in the lipid profile than those in the lean controls.

**Table 2:** Metabolic and inflammatory parameters.

	Lean (n = 16)	Overweight (n = 10)	Obese (n = 19)
Glucose (mg/dL)	84.75 ± 3.00	86.30 ±2.09	87.21 ± 2.81
Insulin (mU/L)	7.64 ± 1.17	12.53 ± 1.70*	12.74 ± 1.84*
Glucose / Insulin ratio	15.51 ± 2.74	8.12 ± 1.25*	8.87 ± 0.96*
HOMA-IR	1.61 ± 0.25	2.67 ± 0.41*	2.82 ± 0.47*
Total cholesterol (mg/dL)	170.06 ± 10.72	188.20 ± 10.77	174.63 ± 4.30
LDL-c (mg/dL)	98.06 ± 8.32	109.20 ± 8.63	109.00± 3.93*
HDL-c (mg/dL)	61.38 ± 4.33	68.30 ± 4.44	51.74 ± 2.38^#^
LDL-c / TC ratio	0.57 ± 0.02	0.58 ± 0.02	0.62 ± 0.01* ^#^
HDL-c / TC ratio	0.37 ± 0.02	0.37 ± 0.03	0.30 ± 0.02* ^#^
LDL-c / HDL-c ratio	1.65 ± 0.13	1.66 ± 0.17	2.19 ± 0.13* ^#^
TG (mg/dL)	62.38 ± 6.45	71.60 ± 11.74	105.84 ± 12.63* ^#^
Log (TG/HDL-c)	-0.01 ± 0.05	-0.01 ± 0.06	0.27 ± 0.07* ^#^
hs-CRP (mg/dL)	0.18 ± 0.02	0.39 ± 0.08*	0.36 ± 0.05*
MCP-1 (pg/mL)	121.40 ± 6.56	117.38 ± 6.21	151.53 ± 11.28^#^
TNF-α (pg/mL)	1.21 ± 0.11	1.82 ± 0.25*	1.76 ± 0.15*
IL-6 (pg/mL)	1.25 ± 0.42	0.94 ± 0.10	2.28 ± 1.08
Leptin (ng/mL)	5.96 ± 0.84	17.89 ± 3.61*	14.84 ± 1.29*
Adiponectin (μg/ml)	6.44 ± 0.93	4.60 ± 0.80	4.91 ± 0.85
PAI-1 (ng/ml)	2.87 ± 0.45	4.60 ± 0.50	5.83 ± 0.64*
FBG (μg /ml)	4.56 ± 0.17	4.96 ± 0.18*	5.00 ± 0.10*

Values are given as the mean ± SEM. Abbreviations: FBG, fasting blood glucose; HDL-c: high density lipoprotein-cholesterol; HOMA-IR, homeostasis model assessment for insulin resistance index; hs-CRP, high sensitivity C-reactive protein; IL-6, interleukin-6; LDL-c, low density lipoprotein-cholesterol; MCP-1, monocyte chemoattractant protein-1; PAI-1, plasminogen activator inhibitor-1; TC, total cholesterol; TG, triglycerides; TNF-α, tumor necrosis factor-α.

* P＜0.05, differences vs. normal weight group;

# P＜0.05, differences vs. overweight group.

The obese children also had significantly higher hs-CRP, TNF-α, and MCP-1 levels than those in the lean controls (Table 2); this finding indicated a pro-inflammatory state in the obese children. The IL-6 level was higher in the obese children, but the difference was not significant. Except for MCP-1, these inflammatory markers were not lower among the overweight group than the obese group. The plasma levels of PAI-1 and leptin were significantly higher in the obese subjects than in the controls. The adiponectin level was lower in the obese group, but the difference was not significant. Moreover, the leptin level was higher, but the adiponectin level was lower in the overweight subjects than in the obese subjects; however, these differences were not significant.

### Correlations among Clinical Data, Metabolic, and Inflammatory Markers

A correlation analysis was performed for all lipid profiles, metabolic and inflammatory markers (Table 3). First, BMI percentile was positively correlated with insulin resistance (fasting insulin and HOMA-IR), lipid profiles (LDL-c, TG and TC), and inflammatory markers (hs-CRP, MCP-1, TNF-α, PAI-1, and leptin) but negatively correlated with adiponectin level. Second, fasting insulin level was positively correlated with BMI percentile, LDL-c and TG levels as well as the levels of the inflammatory markers leptin and PAI-1 but negatively correlated with the HDL-c/TC and adiponectin levels that were measured as part of the lipid profile. In the lipid profiles, the TG and LDL-c levels were positively correlated with the inflammatory markers IL-6 and hs-CRP, respectively. However, the HDL-c level was negatively correlated with the IL-6 level. Furthermore, the level of leptin (an adipocytokine) was positively correlated with insulin resistance (fasting glucose level, fasting insulin level and HOMA-IR), lipid profiles (LDL-c, TG and TC levels), and inflammatory markers (hs-CRP, MCP-1, TNF-α, and PAI-1 levels) but negatively correlated with the adiponectin level. Meanwhile, the level of adiponectin (another adipocytokine) was negatively correlated with insulin resistance (fasting insulin level and HOMA-IR), lipid profiles (LDL-c and TG levels), and inflammatory markers (MCP-1 and PAI-1 levels).

**Table 3:** The correlations between the clinical data and the metabolic and inflammatory markers.

p ^r^	BMI (%)	Glucose	Insulin	HOMA-IR	HDL-c	LDL-c	TG	TC	HDL-c / TC	hs-CRP	MCP-1	TNF-α	IL6	Leptin	Adiponectin	PAI-1	FBG
BMI (%)		0.212	***0*.*441***	***0*.*471***	-0.124	***0*.*418***	***0*.*334***	***0*.*286***	***-0*.*419***	***0*.*450***	***0*.*440***	***0*.*512***	0.232	***0*.*683***	***-0*.*295***	***0*.*565***	0.184
Glucose	0.139		0.067	0.248	0.249	0.254	-0.192	0.225	-0.035	0.132	0.164	0.016	-0.134	***0*.*351***	-0.112	0.180	-0.076
Insulin	0.001	0.645		***0*.*974***	-0.096	***0*.*293***	***0*.*429***	0.221	***-0*.*343***	0.250	0.170	0.159	0.035	***0*.*529***	***-0*.*341***	***0*.*461***	0.197
HOMA-IR	0.001	0.083	1.03E-32		-0.018	***0*.*329***	***0*.*372***	0.257	***-0*.*313***	0.257	0.176	0.182	0.025	***0*.*601***	***-0*.*372***	***0*.*501***	0.198
HDL-c	0.390	0.081	0.507	0.900		0.141	***-0*.*421***	***0*.*456***	***0*.*626***	-0.156	-0.039	-0.173	***-0*.*302***	0.211	-0.028	0.092	-0.098
LDL-c	0.003	0.075	0.039	0.020	0.329		***0*.*295***	***0*.*903***	***-0*.*621***	***0*.*334***	0.249	0.056	0.169	***0*.*477***	***-0*.*289***	***0*.*502***	0.140
TG	0.018	0.181	0.002	0.008	0.002	0.038		0.198	***-0*.*602***	0.267	0.203	0.198	***0*.*465***	***0*.*332***	***-0*.*439***	***0*.*548***	0.273
TC	0.044	0.117	0.123	0.071	0.001	3.29E-19	0.167		***-0*.*330***	0.249	0.152	-0.053	0.084	***0*.*454***	-0.232	***0*.*491***	0.087
HDL-c / TC	0.002	0.812	0.015	0.027	1.19E-06	1.47E-06	3.83E-06	0.019		***-0*.*428***	-0.216	-0.174	***-0*.*415***	-0.214	0.202	***-0*.*357***	-0.251
hsCRP	0.002	0.399	0.106	0.097	0.319	0.029	0.083	0.108	0.004		0.187	***0*.*408***	***0*.*687***	***0*.*350***	-0.144	0.301	***0*.*347***
MCP-1	0.001	0.255	0.238	0.222	0.789	0.081	0.158	0.291	0.132	0.231		0.257	0.166	***0*.*353***	***-0*.*318***	0.243	0.131
TNF-α	1.45E-04	0.912	0.270	0.207	0.230	0.697	0.167	0.712	0.226	0.007	0.072		***0*.*326***	***0*.*385***	-0.179	***0*.*351***	-0.005
IL6	0.105	0.353	0.811	0.865	0.033	0.240	0.001	0.563	0.003	3.73E-07	0.248	0.021		0.124	-0.040	***0*.*308***	***0*.*454***
Leptin	4.64E-08	0.013	7.91E-05	3.94E-06	0.140	4.59E-04	0.018	0.001	0.135	0.021	0.012	0.006	0.392		***-0*.*533***	***0*.*626***	0.121
Adiponectin	0.038	0.438	0.015	0.008	0.847	0.042	0.001	0.105	0.159	0.356	0.024	0.213	0.785	6.66E-05		***-0*.*402***	0.079
PAI-1	0.000	0.225	0.001	0.000	0.537	0.000	0.000	0.000	0.014	0.059	0.100	0.015	0.035	0.000	0.005		0.231
FBG	0.202	0.598	0.171	0.167	0.498	0.332	0.055	0.549	0.079	0.022	0.366	0.970	0.001	0.402	0.586	0.117	

The lower left portion of the table shows the p-values; the upper right area shows the positive or negative correlation coefficients, and p-values < 0.05 is denoted in bold and italic. Abbreviations: FBG, fasting blood glucose; HDL-c: high density lipoprotein-cholesterol; HOMA-IR, homeostasis model assessment for insulin resistance index; hs-CRP, high-sensitivity C-reactive protein; IL-6, interleukin-6; LDL-c, low density lipoprotein-cholesterol; MCP-1, monocyte chemoattractant protein-1; PAI-1, plasminogen activator inhibitor-1; TC, total cholesterol; TG, triglycerides; TNF-α, tumor necrosis factor-α.

We then applied a factor analysis using the principal component method with a Varimax rotation to examine whether fasting insulin level was clustered with metabolic risk factors among obese children. The variables included in the factor analysis were BMI percentile, glucose, LDL-c/HDL-c ratio, HDL-c/TC ratio, LDL-c/TC ratio, TC/HDL-c ratio, hs-CRP, TNF-α, and PAI-1 (Table 4). Three-domains were identified that explained 74.08% of the total variance (factor 1: 46.05%; factor 2: 15.38%; factor 3: 12.65%). The first factor was designated the lipid domain, upon which the LDL-c/HDL-c ratio, the LDL-c/TC ratio, and the TC/HDL-c ratio positively loaded and the HDL-c/TC ratio inversely loaded. The second factor was denoted the obesity-inflammatory domain, upon which BMI percentile, hs-CRP, TNF-α, and PAI-1 positively loaded. The third factor was the insulin sensitivity domain, upon which fasting insulin and glucose positively loaded.

**Table 4:** The results of a principal component factor analysis with a Varimax rotation among obese male children.

	Lipid domain	Obesity-inflammatory domain	Insulin sensitivity domain
Variable	Factor 1	Factor 2	Factor 3
BMI percentiles	0.29	**0.74**	0.21
LDL-c / HDL-c ratio	**0.97**	0.19	0.07
HDL-c / TC ratio	**-0.95**	-0.21	-0.04
LDL-c / TC ratio	**0.87**	0.13	0.16
TC/ HDL-c ratio	**0.94**	0.21	0.03
CRP	0.23	**0.58**	0.14
TNF-α	-0.09	**0.83**	-0.23
PAI-1	0.30	**0.62**	0.27
Fasting insulin	0.30	0.16	**0.73**
Glucose	-0.11	0.04	**0.84**
% Total variance (%)	46.05	15.38	12.65
% Cumulative variance	46.05	61.43	74.08

Loadings ≥ 0.5 are in boldface; the abbreviations are described in Tables 1 and 2.

## Discussion

Estrogens affect fat cells directly or through estrogen receptors, and promote fat tissue deposition. Evidence suggests that estrogen increases the differences in adipose tissue depots via subcutaneous lipid accumulation in woman and visceral fat deposition in men [32]. In our considerations, only male children were studied because we did not want to show the effects that sex hormones have on obesity; additional studies are required to test for sex differences.

Recent studies have shown that hypertension and obesity-related metabolic disorders are increasingly prevalent among overweight and obese children [33–36]. In addition, children who gain weight and have neck-related hypertension might be at an increased risk for cardiovascular disease as adults [36–38]. In our study, overweight and obese male had higher systolic and diastolic blood pressures than lean controls, which is similar to the results of Saffari et al. [4]; however, this difference was not significant. We also found that the ΔBP was significantly higher among obese subjects than overweight subjects. Our findings indicated that the diastolic blood pressure in childhood obesity-related hypertension is notable. The cross-sectional nature of our study and its small sample size are limitations for validating this condition. A further cohort study is needed to explore the role of the diastolic pressure among overweight and obese children.

Some earlier studies have suggested that obesity and being overweight are chronic inflammatory diseases that affect the growth and maturation of children and increase their risk of disease during physical activity. Other studies have shown that exercise training positively influences these conditions [21,39]. Measurements of proinflammatory cytokines and oxidative stress in obese children revealed many markers that might contribute to endothelial dysfunction such as higher levels of leptin, resistin, and IL-6 as well as lower levels of adiponectin [30,40]. The visfatin and adiponectin levels in obese children altered the effects of physical activity [41–43]. Certain potential screening tools for risk assessment (e.g., serum hs-CRP, fasting insulin, tumor necrosis factor-α, leptin, and adiponectin levels) have been considered for use among children and adolescents [5,17–20]. However, preliminary evidence indicated that systemic inflammation was not necessarily associated with insulin resistance in obese subjects [8]. According to our data, inflammatory cytokine levels such as hs-CRP, TNF-α, leptin, and PAI-1 were significantly higher in the overweight and obese individuals. Furthermore, these participants had lower adiponectin levels than those of the lean subjects. One article noted that a high leptin-to-adiponectin ratio can be used as a noninvasive predictor of nonalcoholic fatty liver disease among obese adolescents [44]. According to our observations, significantly higher leptin levels and lower adiponectin levels were found in obese compared with those in overweight subjects; these trends correlated with the inflammatory status. A further study could be designed to validate the role of leptin in the inflammation found in these two groups. The insulin resistance in the overweight/obese children was significantly higher than that in the lean control subjects, similar to the findings of Saffari et al. [4]. In the correlation analysis, BMI percentile was positively correlated with insulin resistance (fasting insulin level and HOMA-IR) and inflammatory markers (hs-CRP, MCP-1, TNF-α, PAI-1, and leptin levels) but negatively correlated with the adiponectin level. Moreover, the HOMA-IR score was positively correlated with the leptin and PAI levels but negatively correlated with the adiponectin level. These findings reflected the higher prevalence of metabolic syndrome and inflammation in the overweight/obese children, and a follow-up study would be helpful to measure the levels of adipocytokines and inflammation markers at earlier time points as predictors for the development of overweight.

Overweight and obese adolescents have an increased incidence of high blood lipid levels [45]. High levels of LDL-c and TG, combined with low HDL-c levels, have been found in children with central obesity; these changes are dangerous and they have been correlated with cardiovascular disease in the general population [46–49]. Compared with the lean subjects, the obese group in our study showed increased TG levels and decreased plasma HDL-cholesterol levels; however, similar differences were not significant in the overweight subjects. The correlation analysis further revealed that insulin resistance was positively correlated with TG and LDL-c levels but negatively correlated with HDL-c and TC levels in obese children. In addition, our investigation showed that obesity adversely affected TG and LDL-c concentrations, which were positively correlated with all inflammatory markers (including hs-CRP and PAI-1). Conversely, HDL-c and TC levels were negatively correlated with each other and frequently related to dyslipidemia in childhood obesity, thereby representing a cardiometabolic risk. However, no obvious patterns were observed with regard to the data provided by the overweight children in this study. Based on our results, we found that low-grade inflammation seemed to occur earlier than dyslipidemia among overweight and obese children. However, a future cohort study should be performed to confirm this tendency.

A factor analysis was used to facilitate our understanding of the metabolic, inflammatory, and lipid variables in metabolic syndrome; however, this analysis was affected by diseases, participant characteristics, and specific variables [26,50–52]. Previous research evaluated non-diabetic subjects to determine the risk domains of metabolic syndrome or cardiovascular events using factor analysis [26,50,53]. Previous studies also validated the cardiometabolic risk factors present in non-obese adolescents [54,55]. Of all the variables, we attempted to clarify the contributions of insulin sensitivity, lipids and the inflammation index among obese child. Therefore, we applied this method to identify the factor structures of the metabolic, inflammatory, and lipid variables among obese children. Our results identified three domains with regard to the sample of obese children: lipid, obesity-inflammation, and insulin sensitivity. The lipid domain made a greater contribution than the obesity-inflammation and insulin sensitivity domains among obese participants. The major contribution of this study is that a further dimensionality reduction of these factors could be applied to future predictions and analyses of the diseases in obese child (e.g., high blood pressure, kidney disease, and so on). Previous data had shown that low-grade inflammation and endothelial dysfunction is involved in the pathogenesis of obesity-related hypertension in obese children [56]. Serum LDL-c levels, the TC-to-HDL-c ratio and the LDL-c-to-HDL-c ratio are related to coronary artery disease incidence and mortality [57,58]. The evidence that increased serum PAI-1, interleukin-6 and CRP levels are associated with the development of type 2 diabetes is growing [59,60]. To our knowledge, these physiological domains have not been examined via a factor analysis of the data provided by obese children.

In conclusion, childhood weight requires special early attention because insulin resistance, inflammation and dyslipidemia increase the risk of cardiovascular disease in adults. Leptin, an adipocytokine that is positively correlated with inflammation, might be a strong predictor of overweight status among children. We also found that lipid, obesity-inflammation, and insulin sensitivity domains predominantly exist in the data of obese children. These domains might be applied to predict the outcomes of cardiovascular diseases in the future.

## Supporting Information

S1 Dataset Original dataset of forty-five children in our study. (XLS)

S1 Fig Approval document of the Human Institutional Review Board (IRB; No. 9809–009) for our trial. (TIF)

## References

[1] Nasreddine L, Naja F, Chamieh MC, Adra N, Sibai AM, Hwalla N. Trends in overweight/obesity in Lebanon: evidence from two national cross-sectional surveys (1997 and 2009). BMC Public Health. 2012;12:798 10.1186/1471-2458-12-798 22984791 PMC3527186

[2] Lopez KN, Knudson JD. Obesity: from the agricultural revolution to the contemporary pediatric epidemic. Congenit Heart Dis. 2012;7:189–199. 10.1111/j.1747-0803.2011.00618.x 22304860

[3] Orsi CM, Hale DE, Lynch JL. Pediatric obesity epidemiology. Curr Opin Endocrinol Diabetes Obes. 2011;18:14–22. 10.1097/MED.0b013e3283423de1 21157323

[4] Saffari F, Jalilolghadr S, Esmailzadehha N, Azinfar P. Metabolic syndrome in a sample of the 6- to 16-year-old overweight or obese pediatric population: a comparison of two definitions. Ther Clin Risk Manag. 2012;8:55–63. 10.2147/TCRM.S26673 22346358 PMC3277872

[5] Deboer MD. Obesity, systemic inflammation, and increased risk for cardiovascular disease and diabetes among adolescents: A need for screening tools to target interventions. Nutrition. 2013;29:379–386. 10.1016/j.nut.2012.07.003 23022122 PMC3578702

[6] Vykoukal D, Davies MG. Biology of metabolic syndrome in a vascular patient. Vascular. 2012;20:156–165. 10.1258/vasc.2011.201201 22700582

[7] Da Costa LA, Arora P, García-Bailo B, Karmali M, El-Sohemy A, Badawi A. The association between obesity, cardiometabolic disease biomarkers, and innate immunity-related inflammation in Canadian adults. Diabetes Metab Syndr Obes. 2012;5:347–355. 10.2147/DMSO.S35115 23055759 PMC3468056

[8] Cohen JI, Maayan L, Convit A. Preliminary evidence for obesity-associated insulin resistance in adolescents without elevations of inflammatory cytokines. Diabetol Metab Syndr. 2012;4:26 10.1186/1758-5996-4-26 22682228 PMC3509401

[9] Wasilewska A, Tenderenda E, Taranta-Janusz K, Tobolczyk J, Stypułkowska J. Markers of systemic inflammation in children with hyperuricemia. Acta Paediatr. 2012;101:497–500. 10.1111/j.1651-2227.2011.02582.x 22211844

[10] Breslin WL, Johnston CA, Strohacker K, Carpenter KC, Davidson TR, Moreno JP, et al Obese Mexican American children have elevated MCP-1, TNF-α, monocyte concentration, and dyslipidemia. Pediatrics. 2012;129:e1180–e1186. 10.1542/peds.2011-2477 22473371

[11] Illán-Gómez F, Gonzálvez-Ortega M, Orea-Soler I, Alcaraz-Tafalla MS, Aragón-Alonso A, Pascual-Díaz M, et al Obesity and inflammation: change in adiponectin, C-reactive protein, tumour necrosis factor-alpha and interleukin-6 after bariatric surgery. Obes Surg. 2012;22:950–955. 10.1007/s11695-012-0643-y 22527592

[12] Utsal L, Tillmann V, Zilmer M, Mäestu J, Purge P, Jürimäe J, et al Elevated serum IL-6, IL-8, MCP-1, CRP, and IFN-γ levels in 10- to 11-year-old boys with increased BMI. Horm Res Paediatr. 2012;78:31–39. 10.1159/000339831 22832157

[13] Finucane OM, Reynolds CM, McGillicuddy FC, Roche HM. Insights into the role of macrophage migration inhibitory factor in obesity and insulin resistance. Proc Nutr Soc. 2012;22:1–12. 10.1017/S0029665112000730 22914223

[14] Lutgens E, Binder CJ. Immunology of atherosclerosis. Thromb Haemost. 2011;106: 755–756. 10.1160/TH11-10-0683 22011700

[15] Taube A, Schlich R, Sell H, Eckardt K, Eckel J. Inflammation and metabolic dysfunction: links to cardiovascular diseases. Am J Physiol Heart Circ Physiol. 2012;302:H2148–H2165. 10.1152/ajpheart.00907.2011 22447947

[16] Rana JS, Nieuwdorp M, Jukema JW, Kastelein JJ. Cardiovascular metabolic syndrome—an interplay of, obesity, inflammation, diabetes and coronary heart disease. Diabetes Obes Metab. 2007;9:218–232. 17391148 10.1111/j.1463-1326.2006.00594.x

[17] Aycan Z, Berberoğlu M, Ocal G, Evliyaoglu O, Adiyaman P, Deda G, et al Relationship between plasma leptin, insulin and tumor necrosis factor alpha in obese children. J Pediatr Endocrinol Metab. 2005;18:275–284. 15813606 10.1515/jpem.2005.18.3.275

[18] Brito N, Fonseca M, Dinis I, Mirante A. Metabolic factors in obesity. J Pediatr Endocrinol Metab. 2010;23:97–100. 20432812 10.1515/jpem.2010.23.1-2.97

[19] Pyrzak B, Ruminska M, Popko K, Demkow U. Adiponectin as a biomarker of the metabolic syndrome in children and adolescents. Eur J Med Res. 2010;15:147–151. 21147643 10.1186/2047-783X-15-S2-147 PMC4360280

[20] Panagopoulou P, Galli-Tsinopoulou A, Fleva A, Pavlitou-Tsiontsi E, Vavatsi-Christaki N, Nousia-Arvanitakis S. Adiponectin and insulin resistance in childhood obesity. J Pediatr Gastroenterol Nutr. 2008;47:356–362. 10.1097/MPG.0b013e31817fcb67 18728534

[21] Rubin DA, Hackney AC. Inflammatory cytokines and metabolic risk factors during growth and maturation: influence of physical activity. Med Sport Sci. 2010;55:43–55. 10.1159/000321971 20956859

[22] Garanty-Bogacka B, Syrenicz M, Goral J, Krupa B, Syrenicz J, Walczak M, et al Changes in inflammatory biomarkers after successful lifestyle intervention in obese children. Endokrynol Pol. 2011;62:499–505. 22144215

[23] Hobkirk JP, King RF, Gately P, Pemberton P, Smith A, Barth JH, et al Longitudinal factor analysis reveals a distinct clustering of cardiometabolic improvements during intensive, short-term dietary and exercise intervention in obese children and adolescents. Metab Syndr Relat Disord. 2012;10:20–25. 10.1089/met.2011.0050 21936669

[24] Montero D, Walther G, Perez-Martin A, Roche E, Vinet A. Endothelial dysfunction, inflammation, and oxidative stress in obese children and adolescents: markers and effect of lifestyle intervention. Obes Rev. 2012;13:441–455. 10.1111/j.1467-789X.2011.00956.x 22133012

[25] Miranda PJ, DeFronzo RA, Califf RM, Guyton JR. Metabolic syndrome: definition, pathophysiology, and mechanisms. Am Heart J. 2005;149:33–45. 15660032 10.1016/j.ahj.2004.07.013

[26] Lin MW, Hwu CM, Huang YH, Sheu WH, Shih KC, Chiang FT, et al Directly measured insulin resistance and the assessment of clustered cardiovascular risks in hypertension. Am J Hypertens. 2006;19:1118–1124. 17070421 10.1016/j.amjhyper.2006.04.003

[27] Stelzer I, Zelzer S, Raggam RB, Prüller F, Truschnig-Wilders M, Meinitzer A, et al Link between leptin and interleukin-6 levels in the initial phase of obesity related inflammation. Transl Res. 2012;159:118–124. 10.1016/j.trsl.2011.10.001 22243796

[28] Valle M, Martos R, Gascón F, Cañete R, Zafra MA, Morales R. Low-grade systemic inflammation, hypoadiponectinemia and a high concentration of leptin are present in very young obese children, and correlate with metabolic syndrome. Diabetes Metab. 2005;31:55–62. 15803114 10.1016/s1262-3636(07)70167-2

[29] Abdullah AR, Hasan HA, Raigangar VL. Analysis of the relationship of leptin, high-sensitivity C-reactive protein, adiponectin, insulin, and uric acid to metabolic syndrome in lean, overweight, and obese young females. Metab Syndr Relat Disord. 2009;7:17–22. 10.1089/met.2008.0045 19025443

[30] Cole TJ, Bellizzi MC, Flegal KM, Dietz WH. Establishing a standard definition for child overweight and obesity worldwide: international survey. BMJ. 2000;320:1240–1243. 10797032 10.1136/bmj.320.7244.1240 PMC27365

[31] Friedewald WT, Levy RI, Fredrickson DS. Estimation of the concentration of low-density lipoprotein cholesterol in plasma, without use of the preparative ultracentrifuge. Clin Chem. 1972;18:499–502. 4337382

[32] Palmer BF, Clegg DJ. The sexual dimorphism of obesity. Mol Cell Endocrinol. 2015;402C:113–119. 10.1016/j.mce.2014.11.029 PMC4326001 25578600

[33] Becton LJ, Shatat IF, Flynn JT. Hypertension and obesity: epidemiology, mechanisms and clinical approach. Indian J Pediatr. 2012;79:1056–1061. 10.1007/s12098-012-0777-x 22664863

[34] Flynn JT, Falkner BE. Obesity hypertension in adolescents: epidemiology, evaluation, and management. J Clin Hypertens (Greenwich). 2011;13:323–331. 10.1111/j.1751-7176.2011.00452.x 21545393 PMC8108776

[35] Nafiu OO, Zepeda A, Curcio C, Prasad Y. Association of neck circumference and obesity status with elevated blood pressure in children. J Hum Hypertens. 2014;28:263–268. 10.1038/jhh.2013.93 24088717

[36] Guo X, Li Y, Sun G, Yang Y, Zheng L, Zhang X, et al Prehypertension in children and adolescents: association with body weight and neck circumference. Intern Med. 2012;51:23–27. 22214619 10.2169/internalmedicine.51.6347

[37] Raj M. Obesity and cardiovascular risk in children and adolescents. Indian J Endocrinol Metab. 2012;16:13–19. 10.4103/2230-8210.91176 22276248 PMC3263181

[38] Masuo K, Rakugi H, Ogihara T, Esler MD, Lambert GW. Cardiovascular and renal complications of type 2 diabetes in obesity: role of sympathetic nerve activity and insulin resistance. Curr Diabetes Rev. 2010;6:58–67. 20034369 10.2174/157339910790909396

[39] l'Allemand-Jander D. Clinical diagnosis of metabolic and cardiovascular risks in overweight children: early development of chronic diseases in the obese child. Int J Obes (Lond). 2010;34:S32–S36. 10.1038/ijo.2010.237 21151144

[40] Arrigo T, Chirico V, Salpietro V, Munafò C, Ferraù V, Gitto E, et al High-mobility group protein B1: a new biomarker of metabolic syndrome in obese children. Eur J Endocrinol. 2013;168:631–638. 10.1530/EJE-13-0037 23384711

[41] Mäestu J, Jürimäe J, Jürimäe T. Visfatin and adiponectin levels in children: relationships with physical activity and metabolic parameters. Med Sport Sci. 2010;55:56–68. 10.1159/000321972 20956860

[42] Jürimäe J, Gruodyte R, Saar M, Cicchella A, Stefanelli C, Passariello C, et al Plasma visfatin and adiponectin concentrations in physically active adolescent girls: relationships with insulin sensitivity and body composition variables. J Pediatr Endocrinol Metab. 2011;24:419–425. 21932575 10.1515/jpem.2011.054

[43] Agueda M, Lasa A, Simon E, Ares R, Larrarte E, Labayen I. Association of circulating visfatin concentrations with insulin resistance and low-grade inflammation after dietary energy restriction in Spanish obese non-diabetic women: role of body composition changes. Nutr Metab Cardiovasc Dis. 2012;22:208–214. 10.1016/j.numecd.2010.06.010 20951014

[44] Angın Y, Arslan N, Kuralay F. Leptin-to-adiponectin ratio in obese adolescents with nonalcoholic fatty liver disease. Turk J Pediatr. 2014;56:259–266. 25341597

[45] Gong CD, Wu QL, Chen Z, Zhang D, Zhao ZY, Peng YM. Glycolipid metabolic status of overweight/obese adolescents aged 9- to 15-year-old and the BMI-SDS/BMI cut-off value of predicting dyslipidemiain boys, Shanghai, China: a cross-sectional study. Lipids Health Dis. 2013;12:129 10.1186/1476-511X-12-129 23984682 PMC3766195

[46] Lawlor DA, Benfield L, Logue J, Tilling K, Howe LD, Fraser A, et al Association between general and central adiposity in childhood, and change in these, with cardiovascular risk factors in adolescence: prospective cohort study. BMJ. 2010;341:c6224 10.1136/bmj.c6224 21109577 PMC2992109

[47] Mokha JS, Srinivasan SR, Dasmahapatra P, Fernandez C, Chen W, Xu J, et al Utility of waist-to-height ratio in assessing the status of central obesity and related cardiometabolic risk profile among normal weight and overweight/obese children: the Bogalusa Heart Study. BMC Pediatr. 2010;10:73 10.1186/1471-2431-10-73 20937123 PMC2964659

[48] van Vliet M, Gazendam RP, von Rosenstiel IA, van Zanten AP, Brandjes DP, Beijnen JH, et al Differential impact of impaired fasting glucose versus impaired glucose tolerance on cardiometabolic risk factors in multi-ethnic overweight/obese children. Eur J Pediatr. 2011;170:589–597. 10.1007/s00431-010-1323-3 20960007 PMC3078320

[49] Friedemann C, Heneghan C, Mahtani K, Thompson M, Perera R, Ward AM. Cardiovascular disease risk in healthy children and its association with body mass index: systematic review and meta-analysis. BMJ. 2012;345:e4759 10.1136/bmj.e4759 23015032 PMC3458230

[50] Meigs JB. Invited commentary: insulin resistance syndrome? Syndrome X? Multiple metabolic syndrome? A syndrome at all? Factor analysis reveals patterns in the fabric of correlated metabolic risk factors. Am J Epidemiol. 2000;152:908–911. 11092432 10.1093/aje/152.10.908

[51] Anderson PJ, Critchley JA, Chan JC, Cockram CS, Lee ZS, Thomas GN, et al Factor analysis of the metabolic syndrome: obesity vs insulin resistance as the central abnormality. Int J Obes Relat Metab Disord. 2001;25:1782–1788. 11781758 10.1038/sj.ijo.0801837

[52] Hanley AJ, Festa A, D'Agostino RB Jr, Wagenknecht LE, Savage PJ, Tracy RP, et al Metabolic and inflammation variable clusters and prediction of type 2 diabetes: factor analysis using directly measured insulin sensitivity. Diabetes. 2004;53:1773–1781. 15220201 10.2337/diabetes.53.7.1773

[53] Edwards KL, Burchfiel CM, Sharp DS, Curb JD, Rodriguez BL, Fujimoto WY, et al Factors of the insulin resistance syndrome in nondiabetic and diabetic elderly Japanese-American men. Am J Epidemiol. 1998;147:441–447. 9525530 10.1093/oxfordjournals.aje.a009469

[54] Charlton R, Gravenor MB, Rees A, Knox G, Hill R, Rahman MA, et al Factors associated with low fitness in adolescents—a mixed methods study. BMC Public Health. 2014;14:764 10.1186/1471-2458-14-764 25074589 PMC4132898

[55] LaBresh KA, Ariza AJ, Lazorick S, Furberg RD, Whetstone L, Hobbs C, et al Adoption of cardiovascular risk reduction guidelines: a cluster-randomized trial. Pediatrics. 2014;134:e732–e738. 10.1542/peds.2014-0876 25157013 PMC4144001

[56] Garanty-Bogacka B, Syrenicz M, Syrenicz A, Gebala A, Lulka D, Walczak M. Serum markers of inflammation and endothelial activation in children with obesity-related hypertension. Neuro Endocrinol Lett. 2005;26:242–246. 15990729

[57] Frost PH, Davis BR, Burlando AJ, Curb JD, Guthrie GP Jr, Isaacsohn JL, et al Serum lipids and incidence of coronary heart disease. Findings from the Systolic Hypertension in the Elderly Program (SHEP). Circulation. 1996;94:2381–2388. 8921777 10.1161/01.cir.94.10.2381

[58] Ding D, Li X, Qiu J, Li R, Zhang Y, Su D, et al Serum lipids, apolipoproteins, and mortality among coronary artery disease patients. Biomed Res Int. 2014;2014:709756 10.1155/2014/709756 24982904 PMC4058853

[59] Mertens I, Van Gaal LF. Visceral fat as a determinant of fibrinolysis and hemostasis. Semin Vasc Med. 2005;5:48–55. 15968580 10.1055/s-2005-871741

[60] Wang X, Bao W, Liu J, Ouyang YY, Wang D, Rong S, et al Inflammatory markers and risk of type 2 diabetes: a systematic review and meta-analysis. Diabetes Care. 2013;36:166–175. 10.2337/dc12-0702 23264288 PMC3526249

