# Supplementary figures and images for: Evidence in Obese Children: Contribution of Hyperlipidemia, Obesity-Inflammation, and Insulin Sensitivity
Source: PLoS One. 2015 May 26;10(5):e0125935. doi: 10.1371/journal.pone.0125935 (PMC4444301; doi:10.1371/journal.pone.0125935)

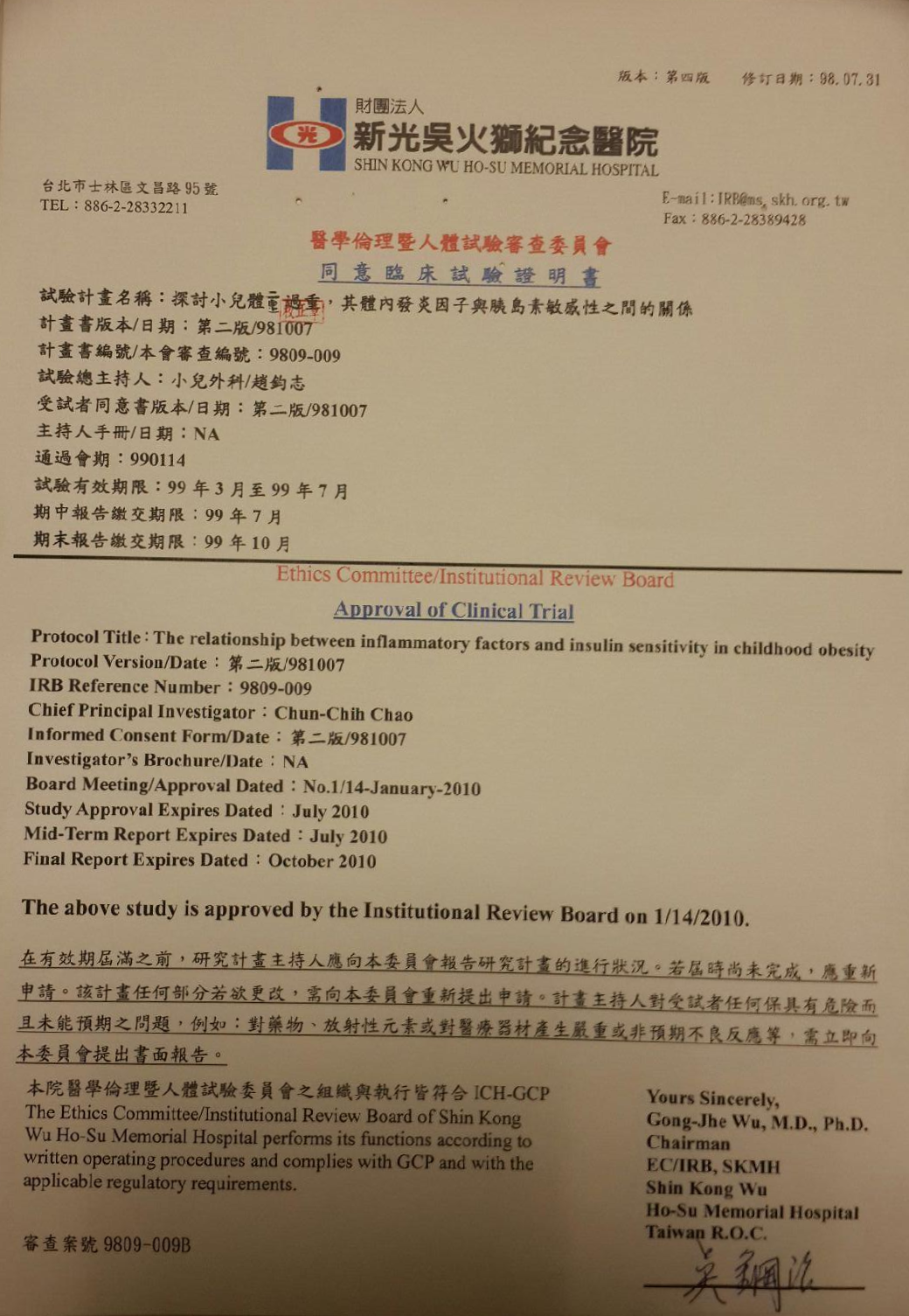

Supplement: S1 Fig — (TIF) [file pone.0125935.s002.tif]
